# Organoarsenic Drugs over Time: The Pharmacokinetics of Roxarsone in Chicken Meat

**DOI:** 10.1289/ehp.124-A150

**Published:** 2016-08-01

**Authors:** Lindsey Konkel

**Affiliations:** Lindsey Konkel is a New Jersey–based journalist who reports on science, health, and the environment.

Although “market basket” studies have revealed the presence of arsenic in packaged chicken,^1^ ^,^ ^2^ little is known about the concentrations of individual arsenic species found in these birds and how they are metabolized. In this issue of *EHP*, researchers determined the levels of arsenic metabolites present in chicken breast after feeding the birds an arsenic-based animal drug called roxarsone.^3^

There are many different species (chemical forms) of arsenic, some more toxic than others. Exposure to inorganic species has been associated with cancer and other adverse health effects in humans.^4^ “We wanted to measure the presence of each arsenic species and describe the elimination kinetics of roxarsone metabolites. Our findings can help guide human exposure assessments,” says senior author X. Chris Le, an environmental chemist at the University of Alberta in Canada.

**Figure:**
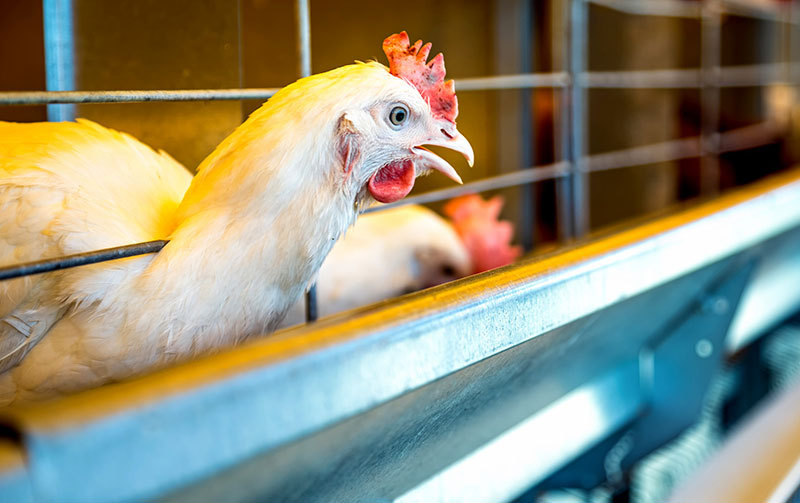
Although roxarsone and other organoarsenic animal drugs are no longer approved for use in U.S. poultry production, some other countries still use them. © Siarhei Simanau/EyeEM

Roxarsone has been used in poultry feed for nearly 60 years to fight parasites and promote growth.^5^ Recent studies have raised concerns over potential human exposure to inorganic arsenic in the livers^6^ and meat^2^ of chicken fed roxarsone. These studies suggested that roxarsone may be partially transformed to inorganic arsenic inside the chicken body as the drug is metabolized. The U.S. Food and Drug Administration (FDA) has since withdrawn approval for any organoarsenic animal drugs to be used in the United States.^7^ However, some countries still allow their use.^8^

Le and colleagues conducted a large feeding trial of 1,600 chickens. For the first month, 800 chickens ate feed supplemented with a dose of roxarsone commonly used in poultry production, while 800 chickens ate a control diet without roxarsone. In the final week of the study, all the chickens received a roxarsone-free diet so that the researchers could see how quickly the drug and its metabolites were eliminated from chicken breast meat once exposure stopped. They collected breast meat samples from chickens at several points over the course of the study, then analyzed the arsenic content.

Concentrations of unmetabolized roxarsone, arsenite (a highly toxic form of inorganic arsenic), and a previously unidentified arsenic species were all significantly higher in chickens fed roxarsone than chickens on the control diet. During the clearance period, when all the chickens were fed a roxarsone-free diet, levels of most arsenic species declined rapidly. However, levels of roxarsone, arsenite, and the unknown arsenic species remained significantly higher in the roxarsone group even 7 days after exposure ceased.^3^

Breast meat from roxarsone-fed chickens had a residual arsenite concentration of 3.1 μg/kg at the end of the study, compared with 0.41 μg/kg in meat from the control chickens. Le and colleagues estimated human daily intake of summed arsenic species from roxarsone-fed chicken would be 0.01 μg/day/kg body weight for a 70-kg adult who ate about 3.5 oz of chicken per day.^3^ That’s much lower, the authors point out, than the World Health Organization provisional tolerable daily intake value for inorganic arsenic of 3 μg/day/kg body weight.^9^

While the findings suggest that meat from organoarsenic-treated chickens may be a relatively minor source of arsenic exposure, there is ongoing debate over what constitutes safe or tolerable levels of arsenic exposure.^10^ “Arsenic appears to be a non-threshold carcinogen, which suggests that any level of exposure corresponds to some level of risk,” says Keeve Nachman, director of the Johns Hopkins Center for a Livable Future Food Production and Public Health Program. “This is a small exposure that is one hundred percent preventable,” he says. Nachman was not involved in the current study.

One of the research team’s next steps will be to investigate arsenic species in poultry waste and track how those forms are taken up and metabolized by plants grown in fields fertilized with the waste. They’ll also look for more detailed mechanistic clues about how a chicken’s body metabolizes arsenic. Le says such clues could provide insights into how the human body metabolizes dietary arsenic.
